# Modifying urban planning to promote child health: A scoping review of reviews

**DOI:** 10.34172/hpp.42995

**Published:** 2024-12-30

**Authors:** Louise Wallerich, Jean Simos, Linda Cambon

**Affiliations:** ^1^University of Bordeaux, INSERM, BPH, U1219, Mérisp/PHARES, Equipe Labellisée Ligue Contre le Cancer, CIC 1401, F-33000 Bordeaux, France; ^2^Institut de Santé Globale, Faculté de Médecine, Université de Genève, Genève, Suisse; ^3^Bordeaux University Hospital, Bordeaux, France; ^4^Bordeaux School of Public Health Prevention Chair, University of Bordeaux, France

**Keywords:** Child, Child health, City planning, Policy, Review

## Abstract

**Background:**

Children’s health is shaped by their physical, natural, and socioeconomic environments. The objective of this study is to identify structural urban planning measures that can positively or negatively impact children’s health. Specifically, we aim to explore how urban planning elements, such as housing, neighborhoods, play areas, and green spaces, influence children’s well-being and health outcomes.

**Methods:**

We conducted a scoping review in accordance with the method developed by Arksey and O’Malley, Levac and colleagues’ methodology advancement, and the Preferred Reporting Items for Systematic reviews and Meta-Analyses (PRISMA) guideline. We explored eight databases, restricting our search to reviews, systematic reviews, and meta-analyses that report on structural measures aimed at modifying urban planning to promote child health.

**Results:**

A total of 41 studies were identified for inclusion in this review. The thematic analysis identified: i) interventions aimed at modifying streets; ii) interventions aimed at modifying play areas; iii) interventions aimed at modifying contact with nature; iv) interventions aimed at modifying deleterious exposures (exposure to tobacco, exposure to school); and v) housing. The second level of analysis enabled us to identify and evaluate the conditions for implementation and effectiveness.

**Conclusion:**

The review highlighted measures that are favorable to children’s health at the level of neighborhood urban planning and questioned the conditions for implementation in a French context.

## Introduction

 As the Geneva Charter^1^ and the Helsinki Declaration on Health in All Policies^2^ underline, the need to act on the physical, natural, and socioeconomic environment to improve people’s health is now widely recognized. Children’s health is rooted in the environment in which they are born, grow up, live, and learn and it shapes their first experiences.^3^ Children are increasingly subject and vulnerable to an environment imposed by adults that they can neither choose nor change. Creating favorable environments for children and adolescents is one of the priorities of the Ottawa Charter for Health Promotion.^4^

 An initial review of the literature on this topic^5^ sought to identify and characterize the environmental determinants of children’s well-being, linked to the built and natural environments and to the child’s socioeconomic environment.^5^ It highlighted five categories of environmental determinants of children’s health, which included factors associated with urban planning, access to green spaces, housing, and neighborhood organization.

 Although there are many recommendations for promoting the well-being of populations in cities, neighborhoods, and organizations, such recommendations nonetheless remain general and are often aimed at adults, without taking into account the specific characteristics and experiences of children.^6-8^ In the absence of clear clarification of the mechanisms by which these determinants and characteristics impact children’s health, the potential levers for action remain difficult to identify and integrate into public policy, despite the call from researchers for greater integration of scientific evidence that links the environment and children’s health into urban policymaking.^3,9-18^

 What needs to be clarified are the practical measures that can be implemented, as well as how to go about doing so. How, for example, can green spaces be distributed around a city to promote children’s well-being? How can we provide children with play facilities that encourage their development? How can we organize cities in a way that encourages children to get around actively and independently? The answer to these questions lies at the heart of a structural approach to health issues, as promoted in the “health in all policies” approach, which describes itself as “an approach to public policies across sectors that systematically takes into account the health implications of decisions, seeks synergies, and avoids harmful health impacts in order to improve population health and health equity”.^2^ It requires empirical work, experimenting with and documenting the solutions implemented or to be implemented. What impact(s) do they have on children? What mechanisms are used to achieve this impact? Under what conditions are these measures implemented, and are they transferable from one context to another, whether cultural, geographical, or organizational?

 We propose to answer these questions through a review of the literature, the aim of which is to characterize the structural urban planning measures likely to have a positive or negative impact on children’s health.

## Material and Methods

###  Study design

 We conducted a scoping review of reviews in accordance with the methodology developed by Arksey & O’Malley,^19^ Levac and colleagues’ methodological advancements,^20^ and the PRISMA guidelines.^21,22^ According to Daudt et al,^23^ “[s]coping studies aim to map the literature on a particular topic or research area and provide an opportunity to identify key concepts; gaps in the research; and types and sources of evidence to inform practice, policymaking, and research”. More specifically, this review addresses the challenges that Antman describes when considering how to summarize and disseminate research results.^24^ Building on the scoping review approach, we chose to scope reviews rather than focus on primary literature: thus it is a scoping review of reviews. This type of review can describe the scope and results of existing research in more detail, in particular areas of study, thus providing a means of summarizing research findings and disseminating them to policymakers.

###  Defining the objectives and inclusion criteria

 The aim of this study is to characterize the structural urban planning measures that are likely to have a positive or negative impact on children’s health. Our research therefore asks: What non-health structural measures aimed at modifying urban planning (neighborhoods, housing, play areas, and green spaces) have an impact on children’s health? What are the conditions for the effectiveness of these measures? What are the conditions for implementing these measures?

 Our inclusion criteria were identified using the Population-phenomena of Interest-Context-Study design (PICOS) framework, adapted from the Population-Intervention-Comparison-Outcomes (PICO) framework.^25^ These criteria are as follows: (*i*) The population of interest is children, defined as anyone under the age of 12 or enrolled in a primary school. The children must have a fixed place of residence and not have any particular diagnosed illnesses or disabilities. Also included in our review is the close circle on which the child depends (parents, carers, etc). (*ii*) The phenomena of interest are structural measures aimed at modifying urban planning. For this review, structural measures correspond to policies, programs, or interventions that modify urban design. Urban design includes the following determinants: housing, neighborhoods, play areas, and green spaces. (*iii*) The results must relate to the health, well-being, or development of the child or to a change in urban design that is favorable to the child. The measures may have been put in place at local, regional, or national levels. Contexts involving specific determinants were excluded. (*iv*) We included three types of design: reviews, systematic reviews, and meta-analyses. Articles had to be published in French or English between 2013 and 2023. Table 1 shows a summary of the inclusion and exclusion criteria.

**Table 1:** Inclusion and exclusion criteria

**Inclusion criteria **	**Exclusion criteria **
**Population**	
Child (under 12 or in a primary school) Close relatives of the child	Child with a diagnosed illness or disability Homeless child Teenager Adult
**Phenomena of interest**	
Structural measures to modify urban planning	Individual measures focusing on the child or his/her parent Measures aimed at modifying determinants other than urban planning Health measures (e.g., vaccination, screening) School health programs (e.g., school oral health program, school nutrition)
**Outcomes**	
The child’s health or a change in urban planning to benefit the child	
**Design**	
Reviews, systematic reviews, and meta-analysis	Other designs
**Context**	
Local, regional, or national level Inclusion of crisis situations: Covid, financial crisis, climate crisis	Specific context involving specific determinants
**Period**	
2013-2023	Before 2013

**Figure 1:**
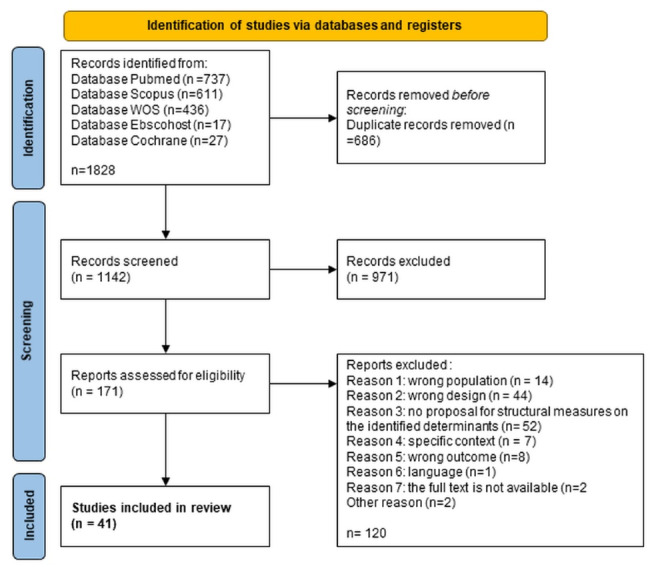
PRISMA flowchart of study selection process

**Figure 2:**
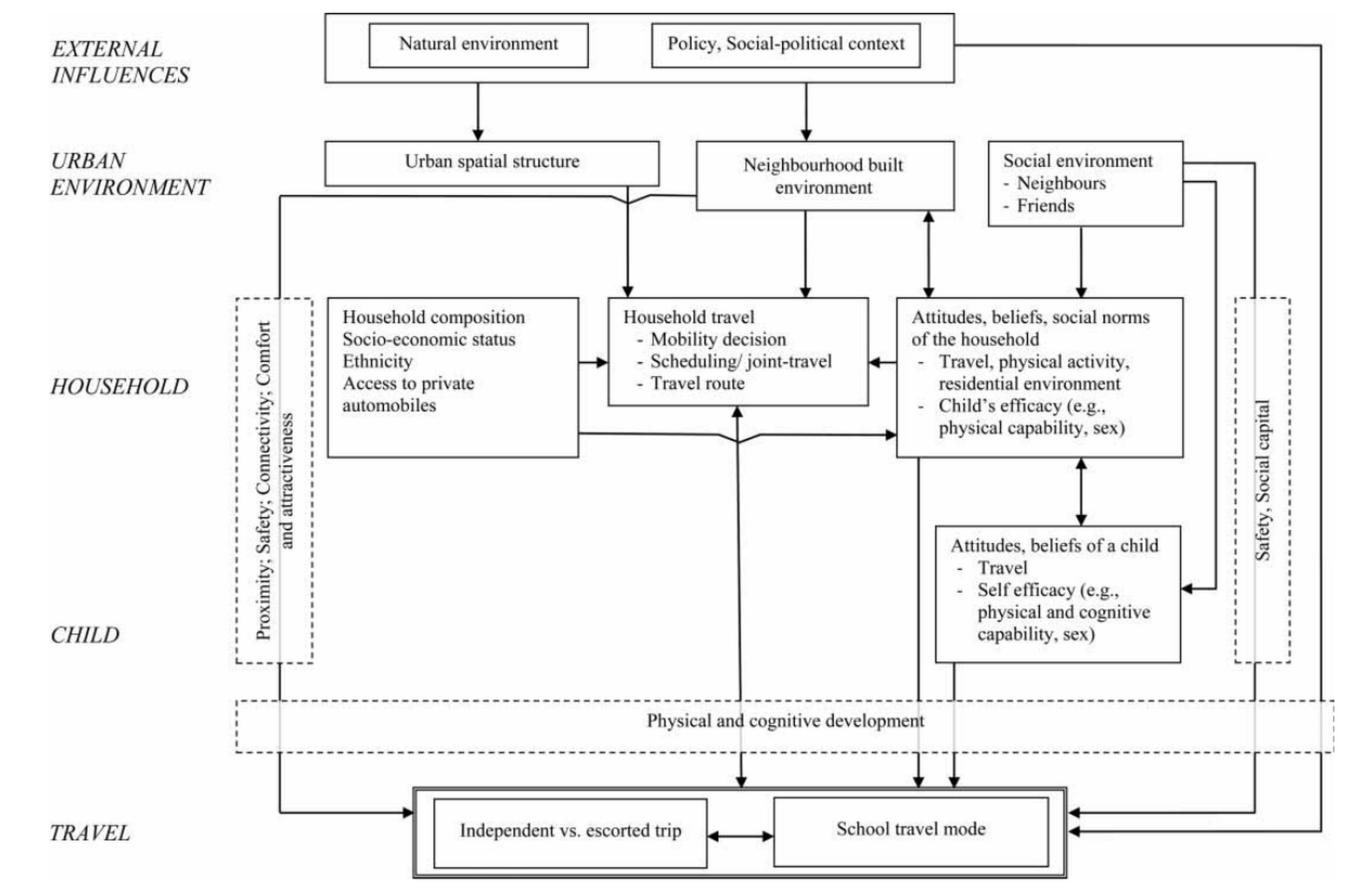
A behavioural model of school transportation^81^

**Figure 3:**
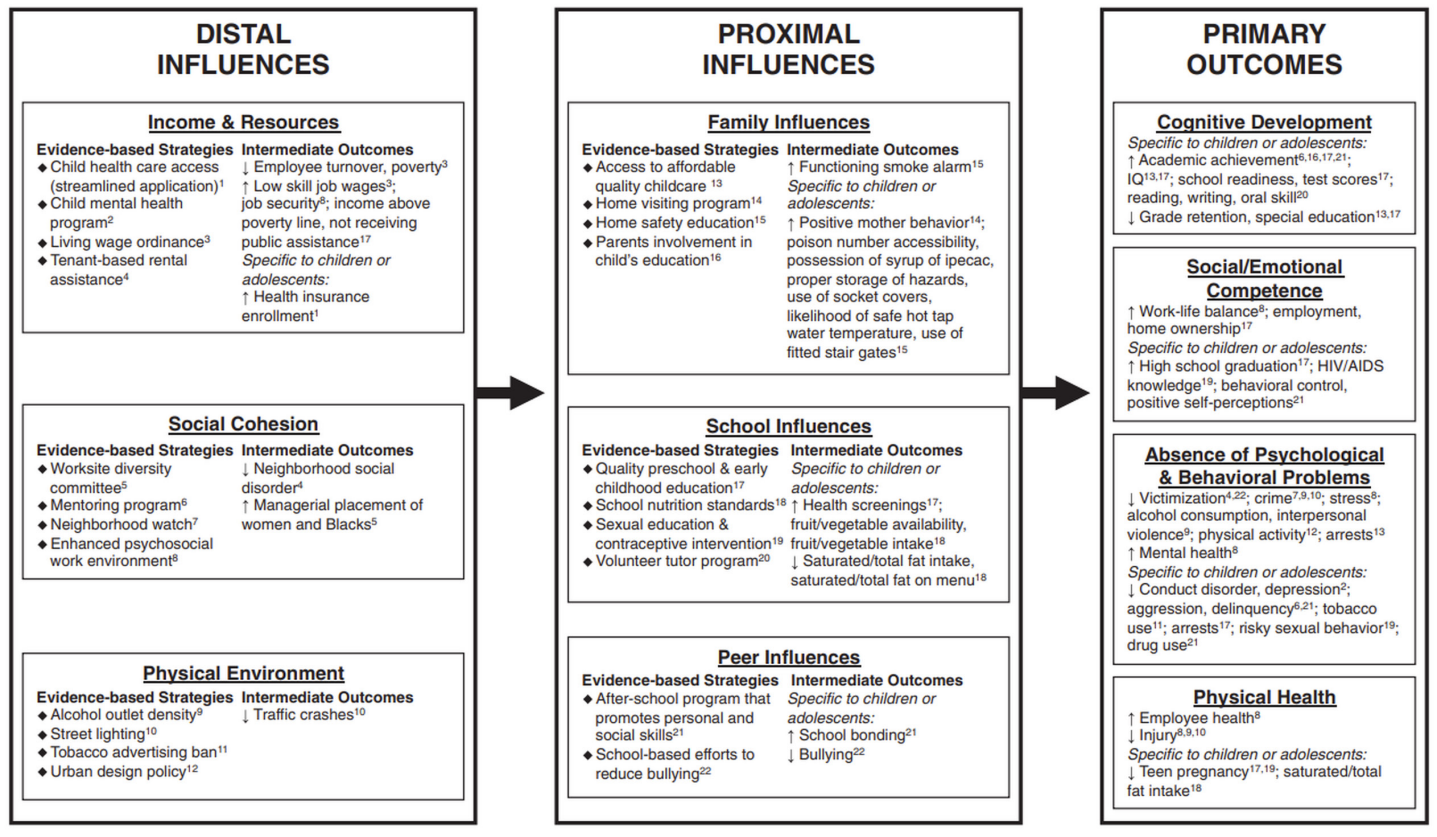
An illustration of combining efficacious community strategies to improve child health and development^33^

**Figure 4:**
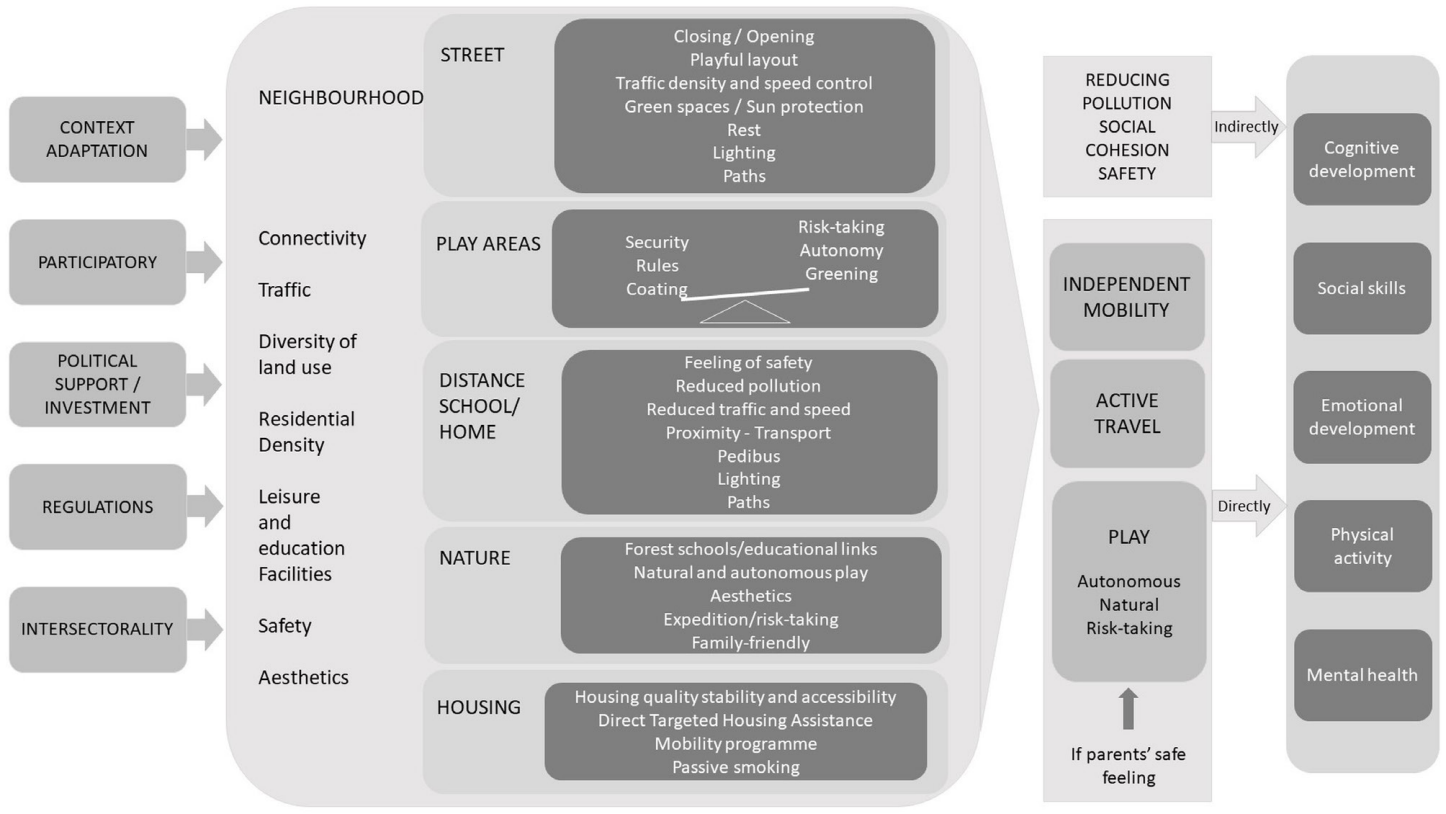
Overview of child-friendly neighbourhood

###  Identification-Search strategy

 To ensure the implementation of the principle of pluralism, the search strategy was developed incorporating eight databases, thus maximizing its coverage of work in relevant disciplines: Scopus, Web of Science, PubMed, SocINDEX, GreenFile, APA PsycInfo, EconLit, and Cochrane. We chose these databases because they are comprehensive and include multidisciplinary journals. Search algorithms were developed for each database with the help of the university librarian and co-authors. Articles were identified during a preliminary exploratory search and nine articles were selected to form the crux of our sample.^26-34^ The algorithms combined three approaches in line with the research questions and inclusion criteria: (*i*) focused on urban planning; (*ii*) focused on the notion of action, modification, measurement, or policy; and (*iii*) focused on child health.

###  Data selection, extraction and analysis

 Selection: Search results were imported into the *Covidence* toolkit for screening. Duplicate records were removed. Study screening and selection was undertaken in two stages. Firstly, title and abstract screening was carried out by one reviewer (LW), after which, at the full-text screening stage, studies were required to meet the eligibility criteria described in Table 1. Records were screened for eligibility independently by two authors (LC, LW). Reasons for excluding articles at stage two are reported in Figure 1.

 Extraction: Study characteristics were independently extracted by each author using an Excel spreadsheet developed by the authors for the purposes of standardized data extraction. Data extracted included: date published; journal of publication; discipline (affiliation of first and last authors); design (review, systematic review, meta-analysis); objective of the article; search method and strategy (databases, inclusion and exclusion criteria, analysis techniques); type of data collected; and identified limitations. In order to answer the research question, the following data were also analyzed: the context; the description of the measure; the assessment criteria for the measure; the effects of the measure; and any other relevant additional information.

 Analysis and synthesis of results: A thematic analysis was conducted using Nvivo® software. The aim was to answer the following questions: (*i*) What type(s) of structural measures aimed at modifying urban planning have an impact on children’s health? (*ii*) What are the conditions for the effectiveness of these measures? (*iii*) What are the conditions for implementing these measures? We deployed a narrative synthesis approach^35^ to summarize our results.

 Positionality and reflexivity: The current review adopts a critical realist perspective.^36-38^ The authors met regularly throughout the review, extraction, and analysis process to discuss the review stages, progress, and team reflections.

## Results

###  Study selection

 A total of 1828 records were identified in database searches in March 2023. Following a screening stage, 41 studies were identified for inclusion in this review. The screening process is illustrated in a PRISMA flow diagram in Figure 1 which sets out the predefined reasons why studies were excluded at the full-text screening stage.

###  Characteristics of included studies

 The articles included were published in journals from various disciplines: public health journals such as the *International Journal of Environmental Research and Public Health*^31,32,34,39-44^ and* BMC Public Health*,^45-48^pediatric health journals such as *BMC Pediatrics,*^33^social science journals such as *Social Sciences*,^49^ and specialist urban planning journals such as the *Journal of Transport and Health*^50,51^ and the *Journal of Urban Health.*^52^Most of the included articles were published after 2018. Of those studies included, 25 were systematic reviews, 15 referred to other types of review, including one scoping review, three narrative reviews, two realist reviews, one rapid review, one mini review, and one meta-analysis.

###  Analysis of measures and interventions and their impact conditions

 The articles identified measures and interventions that we aim to analyze from different angles. They report on neighborhood characteristics that have a direct influence on children’s health and experience (e.g., traffic), which are intervention levers. They also report on interventions, programs, and policies that have been tried, tested, and implemented to the end of improving children’s health (e.g., street closures and the Safe Routes Program). Finally, they provide an analysis of the conditions, costs, and effectiveness of the interventions.

 In the first part of this article, we will describe the characteristics of the interventions, programs, and policies that have been tested according to the five categories of determinants identified. We will then perform a cross-sectional analysis of the conditions required and the evaluations of these interventions.

###  Thematic description of interventions

 We have classified the interventions according to the determinant of the targeted urban development, thus identifying five categories of measures/policies: (*i*) interventions aimed at modifying streets; (*ii*) interventions aimed at modifying play areas; (*iii*) interventions aimed at modifying contact with nature; (*iv*) interventions aimed at modifying deleterious exposures (exposure to tobacco, exposure to adverse school environment); (*v*) housing. Articles exploring a combination of determinants are also presented.

####  Interventions to modify streets

 Streets are at the heart of many interventions, as they are not only conducive to active travel but also relate to children’s independent mobility (CIM).

 Three facilities have been identified by Ortegon-Sanchez et al^31^: street closures; opening up streets for wider uses; and technological developments to encourage active travel. Street closures create safe opportunities for outdoor play by increasing the availability or proximity of public spaces, enhancing the perception of safety from traffic and crime, reducing traffic, and promoting social support. These interventions are temporary and repeated.^31^ According to Umstattd et al, “Play Streets” initiatives strengthen community belonging and can increase physical activity.^48^ Streetscaping aims to encourage community use of spaces and outdoor play for children by modifying pedestrian infrastructure, such as decorated footpaths and car-free spaces, to create shared play surfaces in residential areas.^31^ Technological interventions encourage active travel to school (ATS) by temporarily modifying the environment around the school in a fun way. For example, the “Beat the Street” initiative encourages children to scan music sensors around the school, earning points to increase active travel.^53^

 The home-to-school commute receives particular exploration, with focus on its encouragement of ATS. By adopting an active mode of transportation from childhood, children develop navigation, road safety, and risk management skills, while also fostering decision-making and social interaction. These active journeys are also positively linked to mental well-being and have ripple effects on other environmental determinants of children’s health, such as reducing noise and air pollution, mitigating climate change, and alleviating traffic congestion.

 The “Active Living By Design” ecological model outlines five strategies (“The 5P Strategies”) to promote ATS^54,55^: (*i*) preparation, consisting of partnership-building, funding, training, and data collection; (*ii*) promotion through an advocacy strategy with the target population; (*iii*) programming, whereby the intervention is implemented; (*iv*) policy for the creation of favorable environments; and (*v*) a physical project that modifies the built environment in favor of physical activity (e.g., construction of new parks and walking trails, marking of pedestrian crossings and cycle paths).^54^ Villa-Gonzalez et al^55^ demonstrate that only four of the 23 interventions they identify had mobilized all five strategies, making the effectiveness of these interventions uncertain. The Safe Routes to School (SRTS) program implemented in Canada and the United States aims to create a safe environment and opportunities for children to use active travel to get to school, with the overall objective of increasing children’s use of active travel and, consequently, levels of physical activity.^56^ This program, which can be adapted to suit different contexts and populations, combines educational interventions (e.g., skills workshops for cyclists and pedestrians), changes to the built environment (e.g., installation or widening of cycle paths and pedestrian crossings), and legislative measures (e.g., legislation to apply the program across an area). Finally, “walking school bus” initiatives have been evaluated, with the findings highlighting obstacles such as parents’ safety concerns, recruiting volunteers, and management challenges.^57^

 ATS is also studied at the neighborhood level. Ikeda et al^51^ identify three main interrelated urban characteristics that influence ATS. The first of these is a child’s home’s distance from school, which is strongly correlated with the use of ATS. Rothman et al specify that this may be the distance objectivized or declared.^58^ For illustration, the walking threshold distance (i.e., the distance beyond which car use surpasses walking) was 600 m in 2003 in Montreal.^59^ Moreover, this distance has been decreasing over the years. For the same distance, fewer children are actively walking (87% in 1986 compared with 65% in 2006 for a distance of 800m from home to school in Toronto).^60^ The second urban characteristic is street connectivity, which is positively associated with children’s active travel to all destinations. It offers the possibility of more direct and shorter routes.^61^ However, it has also been noted that highly connected streets can be used more by motorized vehicles. Children are thus more exposed to a large volume of high-speed traffic and are less likely to undertake an ATS.^62^ The relationship between connectivity and ATS is therefore conditional. The third characteristic is residential density, i.e., the number of people or housing units in a given area, which is also positively associated with active travel, because housing units are closer to many destinations.^63^ In particular, the diversity of land uses can reduce the distance to destinations and thus create opportunities for active travel by offering a wider range of possible destinations nearby (a neighbor’s home, school, local shop, cultural or sporting activity, etc).^63^ The accessibility and diversity of destinations are recognized as improving active travel among young people.^64–67^ Wangzom et al^44^ stress that ATS is also largely influenced by several factors, including parents’ safety concerns, which are linked to traffic or children’s exposure to dangerous situations. These concerns encourage parents to take their children to school by car. Therefore, the coexistence of a safe route to school and traffic calming measures is associated with an increase in ATS.^68^ Generally speaking, speed limits are a crucial factor in walkability and safety, influencing active travel^69^ as well as limiting children’s exposure to pollution.^50^ Various measures for speed limits in neighborhoods can be grouped into four major categories: percentage/number of roads with a high speed limit (generally over 50 km/h); perceived safe traffic speed (generally under 50 km/h); perceived speeding; and the use of traffic-calming measures.^69^ In addition, An et al^50^ propose interventions to limit children’s exposure to pollution when travelling in this manner, such as planning transport and walking routes to and from school on the basis of pollution levels and planting trees and hedges on the roads around the school.

 CIM is defined as “the freedom of children to move around their neighborhood or town without adult supervision”.^70^ It is a major factor in active travel.^71^ CIM correlates with various health benefits, including cognitive development through social and environmental experiences. Additionally, spending time together enhances the development of social skills. Conversely, reduced independent mobility in children is linked to increased feelings of loneliness. Marzi et al^71^ appraise neighborhood characteristics linked to CIM: (*i*) heavy car traffic and few play areas discourage independent mobility and affect active travel^72,73^; (*ii*) access to organized leisure activities determines CIM, as children are often driven to activities outside their neighborhood^73^; (*iii*) mothers’ perception of social danger and traffic around the school reduces independent active travel^74,75^; and (*iv*) rising crime rates, heavy urbanization, and long distances to school lead parents to limit CIM by banning children from the road^73,76^ Reed et al^43^ show that the use of outdoor trails could increase children’s active movement, physical activity,^43,77-79^ and exposure to green spaces. Reed et al identify a number of conditions that will make it easier for children to use paths, in particular their proximity, access, attractive landscaping, lighting, and real and perceived safety.^43^ Potential barriers to the use of paths include traffic congestion, lack of path access opportunities, cost, lack of transportation, lack of path routes, and territorial inequalities.^80^

 Mitra^81^ summarizes all of these data and proposes a conceptual framework that might be used when studying the school transport behavior of children and young people (see Figure 2).

####  Interventions aimed at modifying play areas

 Play is key to a child’s development and their relationship with the world, therefore performing a central role in the development of their health. Play spaces and their relationship to health have been studied in a number of ways by various authors and have been grouped according to their location: playgrounds; play in nature; play at school; and play in the neighborhood/outdoor free play (OFP). It is worth noting that most authors have studied play essentially from the point of view of safety and physical activity.

 With regard to play areas, Richmond et al^82^ list interventions, programs, and policies aimed at preventing playground injuries in children under 18. The interventions studied are aimed at: (*i*) reducing risky behavior on playgrounds; (*ii*) increasing the surveillance of playgrounds (which does not lead to a significant reduction in injuries or risky behavior on playgrounds); (*iii*) modifying the surfacing, height, and dangerous features of playgrounds; and (*iv*) drawing attention to and reducing the dangers of playgrounds.^82^

 Suggestions for improvement include adopting nature-based play areas, comprising of environments devoid of play equipment and structures. In addition to the benefits associated with outdoor play, this would reduce the obstacles to compliance with playground safety standards. This finding is corroborated by Tremblay et al^83^ and Brussoni et al^84^ who highlight the considerable health benefits of playing in nature, including more independent play.^83,84^ Indeed, much of the developmental value that children derive from play is due to its intrinsic “unpredictability, spontaneity, aimlessness and lack of personal control, rather than directly from its content”.^85^ Playing in nature has positive effects on physical activity, cognitive development (through play, learning, and creativity), and social and emotional development.^86^ Play in nature generally involves free play and interaction with natural elements such as trees, sand, water, and vegetation. Dankiw et al^86^ draws our attention to the unstructured aspect of play, which allows for greater physical activity but above all allows for so-called imaginative play, which has an impact on cognitive development (development of complex thinking skills) and social development.^86^ When it comes to playing in urban parks, the characteristics of the park influence whether children come and use it. The main features considered relevant in a park, according to the perceptions of those accompanying the children, are: safety, the possibility of outdoor activity (facilities allowing various activities), the presence of green spaces, proximity and the presence of rest areas.^42^ Proximity to the park is not a decisive factor: parents will prefer a park that is further away but better equipped to meet the needs of the whole family.^87^ However, the proximity of parks and play areas to the home or daily destinations encourages OFP among young children.^52^

 As far as OFP is concerned, the authors of the studies consulted are particularly interested in the characteristics of the neighborhood. Gemmell et al grouped neighborhoods into three main interdependent themes: “space for play”; “routes”; and “social environments”.^52^ The availability, accessibility, and acceptability of play spaces in neighborhoods are influenced by the combined characteristics of the spaces, the routes, and the social environment of the neighborhood.^52^ Play areas in neighborhoods, green spaces, car and pedestrian traffic, and neighborhood design that facilitates social links with neighbors encourage OFP for young children. These results are consistent with the findings of Lambert et al, who illustrate an increase in playing opportunities in “dense urban neighbourhoods with traffic calming elements, such as cul-de-sacs that remain permeable to cyclists and pedestrians, speed limits of 15 km/h, and elements, such as benches and trees, that encourage chance encounters and conversations”.^41^ Wray et al confirmed these results by emphasizing that integrating natural and playful elements into outdoor spaces can be an effective way of improving physical activity and social cohesion.^88^

 With regard to play at school during break time, Jerebine et al produced a socioecological model of the risk and safety factors that influence children’s active play at school at five levels (societal, political and institutional, physical environment, interpersonal, and individual).^89,90^ For example, the nature and impact of the rules that are in place in a playground are decided at the political and institutional level. Children perceive these rules as a concern for their physical safety on the part of adults. Restrictions that make playtime boring thus contribute to social conflict, inappropriate behavior, and unequal access to play opportunities. According to Jerebine et al, such perceptions of restrictions could produce a negative feedback effect: by increasing safety concerns and the perceived and real risks of injury in schools, rules and restrictions end up being tightened.^89^ Jerebine et al therefore recommend fostering a culture of risk tolerance in schools and renegotiating the rules of the playground.^89^ These factors limit the school’s ability to provide a recreational environment that truly encourages active play.^90^ This could explain, in part, the inconclusive results on physical activity of interventions in school grounds that consist of providing playground equipment or playground markings.^91^

####  Interventions designed to modify contact with nature

 Nature and natural environments are broadly defined to include living plants and animals, geological processes, and meteorological conditions.^92^

 The review by Barrable and Booth identifies interventions aimed at increasing children’s contact with nature (mainly environmental education programs in educational settings).^93^ Their results suggest that contact with nature is most effective through play, enjoyment, and commitment by the child. The authors emphasize the need for this contact to be sustained through initiatives such as forest schools, nature kindergartens, adventure activities, and expeditions exploring flora and fauna^93^; and they underline the positive influence of this contact with nature on children’s cognitive and behavioral development, as well as on their health.^40,94^ By way of illustration, we can cite the effect on pupils’ cognitive levels through interventions such as teaching outdoors in natural environments.^40^ Some research even highlights the positive effects that merely having sight of green spaces from the classroom can have on pupils’ the cognitive functions and stress levels.^94^

 The effects of green spaces are, of course, nuanced, and in particular the characteristics of green spaces need to be considered: for instance, canopy or tree cover better explains results in terms of cognitive performance than other green cover, such as low or herbaceous vegetation. Interventions to improve the physical space of the school environment with vegetation should focus on planting with tall vegetation, such as trees, which may be more cost-effective for health than other types of low vegetation, such as grass. In addition, nearby vegetation can help to mitigate the negative effects of pollution.^40^

 The initiative to plant school grounds was explored by Bikomeye et al^39^ in their examination of experimental studies on how greening school grounds impact measures of physical activity and socioemotional health in children. A school’s outdoor environment is modified with a combination of natural elements (e.g., trees, flowers, sand, water, grass, hills, and bushes) to create more attractive school grounds and improve the quality of children’s play experiences. The results indicated: (*i*) an increase in physical activity amongst children; (*ii*) a reduction in equity gaps in physical activity; (*iii*) more enjoyable opportunities for creative free play, in turn reducing boredom and increasing pupils’ motivation to play; (*iv*) a positive impact on socioemotional health (pro-social behavior, reduced physical and verbal conflict, social support); and (*v*) a positive impact on mental health. Greening school grounds is a promising strategy for reducing health inequalities through access to green spaces for all children, regardless of their place of residence or socioeconomic status, and equal opportunities for play.

####  Interventions to modify deleterious exposures

 A number of publications examined the effects of other environmental determinants on children’s health, such as exposure to tobacco^46,95,96^ and school buildings.^32^

 With regard to exposure to tobacco, Monson and Arsenault^95^ demonstrate that legislative restrictions on smoking in public places do not “displace” smoking into the home; overall, they reduce levels of passive smoking in children. Restrictions on smoking at home have increased since the legislative bans came into force, helping to normalize negative perceptions of smoking and passive smoking. These results were corroborated to some extent by the meta-analysis conducted by Nanninga et al.^46^ In addition, Mlinarić et al^96^ clarify the mechanisms that influence the implementation of smoke-free measures at local level, including building trust, reinforcing priorities, and limiting divergent interests.

 Finally, Fernandes et al^32^ address the issue of the built and natural environment in schools and report three main types of intervention: (*i*) improving indoor air quality by increasing ventilation rates in classrooms; (*ii*) increasing the amount of time children spend outdoors or greening schools; and (*iii*) multi-component interventions aimed at increasing ATS through changes to pedestrian facilities. This work confirms the results observed elsewhere within the school environment.

####  Housing

 The impact of housing on children’s health is complex, multiform, and intergenerational.^34^ Reece distinguished three pathways by which housing directly influences maternal and child health: (*i*) the habitability and quality of housing (exposure to contaminants, insalubrity, poor air quality, overcrowding, poor insulation); (*ii*) the neighborhood environment (crime, lack of resources, environmental degradation, neighborhood isolation, and deterioration of social capital) in particular for disadvantaged neighborhoods; and (*iii*) the stability and accessibility of housing (foreclosure, transience, eviction, and homelessness) in particular for low-income renter households.^34^

 Reece distinguishes three levels of housing interventions.^34^ The first level concerns so-called direct targeted housing assistance. This is a form of intervention, coordinated with social services, care services, and legal assistance, that prioritizes housing for families that include pregnant women and young children. Early evaluations of such interventions in the United States have shown promising results.

 The second level concerns housing mobility programs. These programs offer low-income families living in deprived neighborhoods the opportunity to move to more economically advantageous neighborhoods by helping the families to find homes, financial assistance with moving costs, and support after the move. Evaluations of mobility programs have shown positive outcomes across education, earnings, and health for young children, particularly girls.^34^ These programs aim to combat patterns of racial and economic segregation and exclusion in the housing market.

 The third level consists of community planning and improvement strategies. Neighborhood-based housing interventions involve multi-sector collaboration, advocacy, and action plans for children’s health. The “Best Babies Zone” approach in the US, for example, targets structural determinants of health by aligning resources, building community leadership, and transforming opportunities in education, economic development, and community systems.^97^

####  Combined approaches

 A number of researchers have taken a more holistic approach to the child’s environment, grouping together all the strategies aimed at modifying the child’s environment more globally. Audrey and Batista-Ferrer^18^ and Nordbø et al^98^ examine the evidence linking the built environment and health through the following characteristics: traffic; the installation of facilities or services (public and private leisure facilities, sports facilities, playgrounds, cultural centers, etc); and pedestrian infrastructure. Audrey & Batista-Ferrer particularly report on multi-component interventions with effects studied on physical activity, eating habits, and body mass index.^18^ They conclude that interventions aimed at reducing road accidents and at increasing young people’s ATS seem promising, whereas there is less evidence that interventions on parks and playgrounds increase their use or, for that matter, bring about the supposed effects of their use.^18^ Nordbø et al identify other previously unidentified characteristics, such as neighborhood aesthetics. Since Nordbo and colleagues’ study, a number of studies on neighborhood aesthetics have shown that less favorable aesthetic conditions are associated with a greater number of behavioral and mental health problems.^98-100^

 Jansson et al^101^ study the concept of “Child-environments”, pointing out that the creation of a child-friendly environment refers to the creation of safe, equitable environments with accessible and diversified green and open spaces. The authors also introduce the importance of “Fairness and Inclusion”, defined as equal opportunities of accessing the city’s services and spaces, which therefore address segregation by age, social level, or gender. They detail the elements needed to create these child-friendly environments, highlighting that interdisciplinarity is needed in bringing about change, taking account of the children’s point of view and adapting measures to each context.

 Kormo et al^33^ propose a framework for action, presented in Figure 3, that articulates combined strategies at a neighborhood level to promote children’s health and development. Their 95 guidance notes are designed to guide actors and decision-makers and provide a starting point for future research.

###  Necessary conditions for intervention

 The cross-sectional analysis of the articles included highlights the conditions that are described as necessary or as playing an important facilitative role in the design and implementation of interventions/measures or their positive effects.

####  Brief description of complex interventions

 Some scholars highlight the lack of information on the interventions themselves and the conditions under which they were implemented (duration, context, funding, etc). Such a lack is seen as damaging because it prevents researchers from understanding the results of the evaluation, from generalizing findings, and from devising orienting policies without some difficulty.^31^ For example, Lu et al indicate that only few interventions to promote ATS are based on theory, meaning that the overwhelming majority have no explicit hypotheses about how they work.^102^ However, these interventions are complex in that they combine different strategies that need to be integrated. They therefore require planning, which is rarely described in the articles, yet is necessary for their replication and feasibility.^56^

 Interventions must be adapted to organizational contexts and populations (e.g. organized sport activities, information on services, additional facilities).^31^ Both the resources and the interweaving of activities required for their feasibility need to be reported. In fact, the physical structural conditions of the interventions, the specific characteristics of the location, and the contextual factors are rarely described. This point is echoed by Ortegon-Sanchez et al in their discussion of street-level interventions; they criticize “a lack of description of the specific attributes of the built environment that relate to the characteristics of the street and the contextual area where the intervention was to be implemented (for example, whether it was a residential or mixed-use street, whether it was a local street or a main road, what type of buildings or land uses were in the block, whether it was shaded or unshaded, and so on)”.^31^ Such contextual factors can have a major influence on the results, as shown by Lu et al, who identify that the perceived barriers to ATS can vary depending on the natural environment and conditions.^102^ The sensitivity of the measures to these contexts calls for interventions to be adapted to local needs and requirements, which can only be achieved by a detailed description of the influence they have on the outcome of the intervention.^58,86,103^

 Lastly, these interventions require strong political and financial support, which sometimes determines the positive results obtained, especially as the health outcomes are either indirectly visible or only visible in the long term. However, costs and funding, such as a seed grant for a street closure intervention,^31^ are rarely mentioned, or only marginally mentioned. Political support from the Senate and House of Representatives in the United States has increased funding opportunities to launch and support the SRTS intervention,^56^ and federal legislation has set aside funding for SRTS programs. Nevertheless, as Jacob et al point out, interventions that improve infrastructure and make ATS safer and easier generate societal economic benefits that outweigh the societal costs.^104^ Taking these factors into account is an important lever for advocacy when it comes to disseminating health measures in all policies.

####  Coercive measures

 In some studies, the authors also highlight the political conditions that reinforce the measures put in place. For example, with regard to pollution around schools, An et al stress the need for a regulatory policy on the use of vehicles, a limit on the construction of factories near schools, and city-wide control of vehicle emissions.^50^ Similarly, in the case of CIM and ATS, there is a need to review the measures for locating schools, delimiting catchment areas and providing school transport.^81^ This argues in favor of a resolutely cross-sectoral approach in which measures are not considered in isolation or on a temporary basis, but are integrated into a more comprehensive, shared approach to issues relating to children’s health.

####  Cross-sector approach

 Along these lines, the sectors mobilized by the interventions studied vary. To illustrate this, we can cite: (*i*) urban planning for the shape of roads, the layout of buildings, and the location of schools^50^; (*ii*) the police, by organizing street patrols and enforcing road closures^31^; (*iii*) the transport sector to improve infrastructure^50^; (*iv*) legal protection to help families ensure that their civil and housing rights are respected^34^; and (*v*) education by mobilizing access to nature in the teaching process, etc.^89,90,93^

 As a result, there are many partners. For example, the action plans and policies for the SRTS program involve schools, municipalities and community partners in a bottom-up approach that promotes cultural change at both individual and political levels.

 This cross-sectoral approach is not without its difficulties, as highlighted by Richmond et al, in their study of the implementation of play areas: the lack of coordination, the time devoted to the project, funding, and staffing problems are all obstacles to compliance with and maintenance of safety standards, for example.^82^ Reece et al^34^ stress the need for coordination in the implementation of healthy housing initiatives, in particular through collaboration and knowledge-sharing between the health and non-health sectors.

####  Participatory approach and involvement of local people

 The involvement and needs of the population seem to be a central element in many interventions at different levels.

 For example, the work of Padial-Ruz et al stresses that the needs of the population (children and family members who look after them) and the socioeconomic context of the area in which interventions are implemented must be taken into account when building and/or redeveloping parks.^42^ Reece et al call for the involvement of local people to be systematically considered in public health projects.^34^ They point out that this community participation has historically been at the root of essential social movements that promote public health, environmental justice, and housing.^34^

 Some authors refer to a consultation of the community,^31^ or are even the direct result of the community, such as playstreets where pupils take part in the final design of the streets.^31^ ATS initiatives increasingly involve schools, parents, and the community.^55^ For the SRTS program, parents and pupils are also involved in both planning and implementation processes.^56^ According to Padial-Ruz et al, involving community members in the renovation of play areas can have a positive effect on park use and children’s engagement in physical activities.^42^

 In this sense, Jerebine et al^89^ recommend consulting children and involving them in decisions that affect their play environment. This is confirmed by Rothman et al,^58^ who point out that studies generally focus on parents’ perceptions, thereby overlooking the huge opportunity that children represent in implementing an intervention, such as understanding how it works and its benefits. Rothman et al write: “The muted voice of children is a missed opportunity to inform research and policy from evidence gathered from the group most directly affected by changes in school travel.”^58^

###  Evaluating interventions

 One of the conditions for scaling up measures to promote children’s well-being is to evaluate them. However, on this subject, the research included points to a few limitations and questions. The first issue highlighted is the standardization of evaluation tools and methods (quasi-experimental study with control group and longitudinal study).^31,71,93^ Indeed, connectivity is usually measured or assessed using the spacing between streets, the number of three- or four-way intersections in an area, or the diﬀerence between the distance between streets and pedestrian networks and the Euclidean distance (i.e., “as the crow flies”) between these.^63,105^ Physical activity is also measured in a wide variety of ways, including pedometers, accelerometers, GPS tracking, observation, self-reporting, etc.^31,47,48,55,86,91,98^ Where this becomes more complicated is in the assessment of children’s play behaviors, for example, which are not characterized by a standardized tool. However, as Dankiw et al write, “the development of a such a tool would ensure consistency when evaluating children’s cognitive/play behaviours, enabling the comparison and pooling of research findings to produce a more robust evidence base for academics, health practitioners, educators and policy/decision-makers”.^86^ Discussions on this issue follows the principle of “no controls, no conclusions”.^106^ This helps to explain why structural measures and their evaluation have difficulty in informing public policy, since the results are not considered to be reproducible or generalizable.

 Some authors nevertheless point out the limitations of this vision. For example, on the effects of nature, Vella-Brodrick and Gilowska state that it is not possible to establish cause-and-effect relationships, nor does this type of evaluation provide a clear understanding of the factors that influence the positive effects of nature on cognitive functioning.^94^ Reece adds that public health agencies continue to deploy standardized “tried and true” interventions which do not meet the needs of all populations in all contexts.^34^

## Discussion

###  Summary of findings

 Child-friendly neighborhood development impacts a number of aspects of children’s health and development: cognitive development (e.g., navigation skills, road safety, imagination, problem-solving, risk management); social skills; emotional skills; physical activity; and positive mental health (e.g., happiness, satisfaction, joy). These outcomes result from direct proximal determinants like diverse, unregulated, autonomous play areas, active travel, and independent mobility, which are feasible when parents perceive neighborhoods as safe. Indirect factors include reduced urban pollution, strengthened social cohesion (increased belonging, solidarity, social exchanges), and secured environments (traffic regulation and increased parental presence outside). Five urban attributes crucial for child-friendly neighborhoods are street configuration, the home-school route, play areas, access to nature, and housing. Each attribute includes specific features and conditions that promote independent mobility and play. These attributes are part of broader neighborhood development conditions, such as street connectivity with regulated traffic, diverse land uses that reduce distances between services and homes, and access to nearby leisure and educational facilities. Lastly, the structural conditions necessary for such neighborhoods include political support, investment, regulation, and community involvement (participation of children and parents, cross-sectoral collaboration).

 Based on the above results, we propose Figure 4. This figure summarizes the main findings of the study, structuring them in five levels from right to left. The first level is that of the results of child-friendly neighborhood development measures on children’s health and development. These results are the combined consequence of direct proximal determinants (second level), the development of diversified, unregulated, autonomous and natural play areas, active travel and independent mobility, the latter made possible if parents feel that the areas dedicated to their children are safe. These results are also the consequence of more indirect proximal determinants (pollution, social cohesion, securing of places). The third level is that of the “attributes” of the child-friendly neighborhoods. The review enabled us to identify five urban attributes to which attention should be paid: street configuration, the home-school route, play areas, access to nature, and housing. These conditions and attributes are therefore part of the more general conditions of neighborhood development (level 4). Finally, the fifth level covers the structural conditions necessary for the viability and existence of such neighborhoods, whether these conditions are political in nature, such as support, investment, regulation (including coercive measures), or process-based, through the participation of children and parents in the definition of measures, cross-sectoral reflection, and commitment (education, transport, urban planning, etc), and adaptation to each context. This framework aims to guide decision-makers in creating child-friendly neighborhoods and to assist researchers in identifying effective measures.

 Some authors provide interesting additions on walkability, independent mobility, and ATS. For example, Crawford et al propose a socioecological model for CIM, based on a qualitative study of children and parents.^107^ Their research shows both the effects and interactions between the political and legislative, physical, social, and community, and family and individual (parent and child) environments that determine when, how, and to what extent children benefit from independent mobility.^107^ Moreover, independent mobility seems to depend on the destination chosen and the impact of neighborhood characteristics varies according to that destination. For CIM to parks, they need to be able to access different types and sizes of urban green spaces.^108^ McMillan^109^ suggests that an intervention in the built environment that aims to increase the number of children walking to school could be better achieved by focusing on the mediating factors, defined as factors resulting from the built environment, the “urban form”. These are traffic safety (actual/perceived), neighborhood safety (actual/perceived), and household transport options/distance to school. Similarly, the framework describes moderating factors that influence decisions regarding children’s ATS. The moderating factors are social or cultural norms, parental attitudes, and sociodemographic characteristics. In our model, we did not distinguish between elements according to whether they were mediators or moderators. With regard to the relationship with nature, the positive effects of contact with nature have also been reported by other authors. Welles & Evans state that in a rural environment, levels of proximity to nature moderate the impact of stressful life events on children’s psychological well-being.^110^ Similar results were found in low-income urban neighborhoods where school grounds contained large quantities of plants.^111^

## Limitations

 Our review is not exhaustive. Some articles, although interesting, were not included, even though they were identified in our exploratory search or cited in the references of the articles selected. This is regrettable, as including such articles would enable us to extend and complete our results.

 We were unable to find any specific measures on matters of neighborhood safety and crime. Although research has shown that poorer physical and mental health outcomes are associated with exposure to violence or crime in the neighborhood,^112-115^ we did not identify any measures in this area.

 The search and article selection strategy also faces its own limitations. Firstly, only articles that had been published and reprinted in journals were included, leading to publication bias. In addition, we did not investigate the grey literature. However, this research is very important. The aim of our work is to understand how to guide decision-making. Studying the grey literature would help us to understand and analyze which measures or interventions are brought to the attention of those who take them. We are therefore planning to devote a specific research project to the grey literature in the hope that such research will feed into our thinking on the evaluation of interventions. Some authors have highlighted inconsistencies in the presentation of interventions and a lack of formal evaluation methods in the grey literature in this field.^31,103^ We did not carry out a review of primary scientific evidence (intervention evaluations, for example); instead, given the extent of the existing literature, we relied on published reviews and systematic reviews. Consequently, this analysis may not take very recent studies into account. In addition, the combination of heterogeneous data sources adds value to the results, but it is difficult to summarize their main findings or conclusions. Articles differ in their ability to describe different aspects of these interventions or policies.

###  Knowledge gaps and research avenues

 In addition to presenting the summary framework (Figure 4), this review has enabled us to identify several avenues for further research.

 Firstly, we are looking at the way in which children, their health, and their experiences are viewed. Although we have tried to highlight the salient results identified in reviews of child development, it has to be said that many studies are still very much focused on a vision of health that is reduced to particular individual behaviors (e.g., physical activity), in order to correspond to established individual recommendations (e.g., 60 minutes of activity per day from the WHO) with the aim of reducing the onset of disease (e.g., obesity).^31,39,42,43,45,48,55,56,69,71,81,88,91,102^ This applies to all the determinants studied,^116-119^ including the issue of access to nature, which is studied for the most part from the point of view of physical activity.^120^ This approach is rather simplistic and contradicts the global and positive approach to health described in the Ottawa Charter.^4^ The capacity for self-fulfillment, skills for coping with life’s difficulties, social development, and a sense of security, for example, are not sufficiently emphasized, even though action on structural determinants has multiple and combined effects on physical, mental, and social development. The ambivalence projected by this separation – mental health/physical health orientation – is particularly visible in the issue of playgrounds, where what is at stake is an approach that prioritizes hyper-security in order to avoid accidents versus the development of natural autonomous play conducive to risk-taking, the latter of which benefits children’s development and mental health. Researchers should consider adopting a holistic approach to children’s health that transcends the focus on individual behaviors like physical activity and includes broader determinants such as social development and a sense of security. This approach challenges the reductionist views of health and aligns with the comprehensive health model outlined in the Ottawa Charter.

 Added to this is the adult’s view of the child, and in particular the parents’ view. As described above, the perceived safety of an activity or place is an important issue.^44,89,90,121^ In addition, some authors note a difference in results according to the child’s gender.^39,41,44,52,58,69,71,89,90,98,101^ For example, three studies reported gender differences in the association between speed limits and physical activity, with significant associations being more often reported in girls.^69^ This gender difference may be partly explained by parental factors, as evidence suggests that parents tend to place fewer restrictions on independent play for boys than for girls, while they tend to be more concerned about environmental safety than their children are.^122^ In their study, Brown et al explain that boys seem to benefit from greater mobility and become independent more quickly, whereas girls acquire collective independence by moving around in groups.^123^ The same gendered findings were observed for ATS in relation to parental views on safety.^58^ Parents play a major role in defining and negotiating the territories, choice of modes of transport and independent mobility of their children, and do so in accordance with what they believe a “good parent” should impose. ^124^ We believe that this gendered view of the adult, focused on the child’s physical health and safety, may hinder potential interventions, their mechanisms of action and the effects produced.

 In addition, we are considering how to best evaluate these interventions. How can interventions be studied to ensure that they respond to public policy issues? Most of the interventions studied were cross-sectional studies and randomized controlled trials. These evaluations enabled us to quantify the effect of the intervention, but not to understand how, under what conditions, when, and for whom they produced positive effects. In order to act on the basis of evidence, it is necessary to study whether they are viable, effective, and transferable for potential dissemination. In other words, many of the interventions examined in this review have only been shown to be effective in one population and in one context. This does not mean that an intervention works in all contexts, or under all conditions, in the real world. The majority of studies take a reductionist approach, focusing on the internal validity of interventions. The complexity of health promotion interventions calls for research to be anchored in the pragmatic synthesis/critical realism paradigm.^125^ It mobilizes relevant strategies for evaluating interventions that take account of the context.

 Finally, we question whether these measures take account of territorial and social inequalities in health. Environmental injustices contribute to increasing inequalities in health, and because they persist over time, they are difficult to remedy. It is very difficult to change patterns of spatial injustice in cities, such as the location of polluting factories, dangerous roads or lack of access to parks.^126-128^ Historically, efforts to improve lifestyle behaviors have focused on changing individual behavior,^129^ which is problematic because it assumes that the different population groups can act on an equal basis. For families living in deprived areas where there are structural barriers, such as restrictions on walking, fast food outlets, high levels of traffic, a lack of green spaces or fears about safety, it can be more difficult to adopt a healthy lifestyle. They are more exposed to unhealthy factors and more vulnerable to the negative effects of these factors.^130^ It is now well documented that social conditions in a neighborhood have an impact on children’s health.^3,99,100,131^ Disadvantaged neighborhoods must therefore benefit from proactive policies in all sectors (urban, social, and educational policies) based on a health approach in all policies anchored in local contexts.^130^ Studying the conditions under which the measures that are referenced in the selected studies can be put in place in poor and/or isolated neighborhoods is essential if we wish for this research to guide public policies for the benefit of those who need it most. Researchers should investigate policies that mitigate environmental injustices and improve conditions in disadvantaged neighborhoods. Characteristics of included studies are described in Supplementary file 1.

## Conclusion

 The results of this scoping review have enabled us to draw a number of conclusions. Firstly, we have identified measures that promote children’s health at the neighborhood planning level. This research has highlighted tried and tested initiatives or promising programs and specific neighborhood characteristics that have a direct influence on children’s experience, as well as their impact on children’s health. These impacts are the consequence of direct proximal determinants (play, active movement and independent mobility) or indirect proximal determinants (pollution, safety, and social cohesion), made possible by the attributes and conditions (street configuration, the home-school route, play areas, access to nature, and housing) of child-friendly neighborhoods. From the pragmatic perspective of intervention research in population health, we have also considered the structural conditions for implementing and ensuring the effectiveness of these measures, with a particular focus on their evaluation. Based on the results of this review, we were able to build a framework for understanding what needs to be done to create a child-friendly neighborhood. The main question raised by this review is: what can we learn from this scientific knowledge that can be used in decision-making processes? Our first attempt to answer this question is to propose a summary framework of the attributes of child-friendly neighborhoods, which needs to be developed in response to three challenges. The first is the ability to transfer a planning measure or condition that has been identified as effective to a specific French context. The second is to place the child’s experience at the center of research and decision-making interests and processes. The third challenge is the transfer of knowledge to ensure that the evidence-based solutions produced are applied and are adapted to specific organizational, structural, and cultural contexts. Finally, we have identified avenues of research that, if pursued, promise to develop our knowledge of the various elements reported in this review.

## Competing Interests

 The authors declare that there are no financial or non-financial relationships and activities that interfere the publication of their work.

## Ethical Approval

 Not applicable.

## Supplementary Files

 Supplementary file. Characteristics of the included studies
