# Supplementary material for: Modifying urban planning to promote child health: A scoping review of reviews
Source: Health Promot Perspect. 2024 Dec 30;14(4):319–35. doi: 10.34172/hpp.42995 (PMC11873772; doi:10.34172/hpp.42995)
Supplement: Supplementary file 1 — Supplementary file. Characteristics of the included studies [file hpp-14-319-s001.pdf]

Wallerich et al, **Health Promotion Perspectives.** 2024;14(4):319-S1
doi: 10.34172/hpp.42995
https://hpp.tbzmed.ac.ir

Supplementary file. Characteristics of the included studies

References	Title	Authors	Date	Journals	Design	Objective
18	Healthy urban environments for children and young people: A systematic review of intervention studies	Audrey S, Batista-Ferrer H.	2015	Health & Place	systematic review	The aim of the current systematic review is to examine evidence from intervention studies which involved changes to the urban environment and reported outcomes in relation to health related behaviours, and the physical or mental health outcomes, of children and young people.
31	Shaping Pathways to Child Health: A Systematic Review of Street-Scale Interventions in City Streets. Int J Environ Res Public Health.	Ortegon-Sanchez A, Vaughan L, Christie N, McEachan RRC.	2022	International Journal of Environmental Research and Public Health	systematic review	The aim of this study was to review the evidence of built environment interventions on children's health outcomes. We also aimed to describe key intervention features and quality of studies and provide recommendations for researchers working in this area
32	School-Based Interventions to Support Healthy Indoor and Outdoor Environments for Children: A Systematic Review.	Fernandes A, Ubalde-López M, Yang TC, McEachan RRC, Rashid R, Maitre L, et al	2023	International Journal of Environmental Research and Public Health	systematic review	Specifically, we answer the following research question: what is the effectiveness of interventions with a focus on changing the built and natural school environment, both indoor and outdoor, on (1) improving physical and mental health outcomes such as body composition, asthma symptoms, and cognition, (2) health behaviors such as physical activity and active travel, and (3) levels of exposure to air pollutants, noise, and green spaces in children aged 5 to 12 years?

33	Beyond the clinic: improving child health through evidence-based community development.	Komro KA, Tobler AL, Delisle AL, O'Mara RJ, Wagenaar AC	2013	BMC Pediatrics	theory-driven and structured narrative review of systematic reviews	Our goal is to provide practitioners with a useable summary of a wide-ranging set of scientific literature relevant to community development strategies for child health
34	More Than Shelter: Housing for Urban Maternal and Infant Health.	Reece J.	2021	International Journal of Environmental Research and Public Health	review	The following literature review seeks to understand the deep and complex relationship between urban housing challenges and health, with an emphasis on infant and maternal health.
39	The Impact of Schoolyard Greening on Children's Physical Activity and Socioemotional Health: A Systematic Review of Experimental Studies.	Bikomeye JC, Balza J, Beyer KM	2021	international journal of environmental Research and Public Health	systematic review	we conduct a review of experimental studies of the impact of schoolyard greening on two health outcomes: physical activity (PA) and socioemotional health (SEH). The SEH outcomes consider measures of both children's social interactions and their mental well-being
40	Systematic Review: Neurodevelopmental Benefits of Active/Passive School Exposure to Green and/or Blue Spaces in Children and Adolescents.	Díaz-Martínez F, Sánchez-Sauco MF, Cabrera-Rivera LT, Sánchez CO, Hidalgo-Albadalejo MD, Claudio L, et al.	2023	international Journal of environmental Research and Public Health	review	this review aims to identify the benefits of contact with nature on neurodevelopment in children and adolescents when interacting passively and/or actively in the school environment.
41	What Is the Relationship between the Neighbourhood Built Environment and Time Spent in Outdoor Play? A Systematic Review.	Lambert A, Vlaar J, Herrington S, Brussoni M.	2019	International Journal of Environmental Research and Public Health	systematic review	The objective of this systematic review is to examine the relationship between physical characteristics of the neighbourhood built environment and the time children and adolescents spend in outdoor play.

42	Determining Factors in the Use of Urban Parks That Influence the Practice of Physical Activity in Children: A Systematic Review	Padial-Ruz R, Puga-González ME, Céspedes-Jiménez Á, Cabello-Manrique D	2021	International Journal of Envrionmental Research and Public Health	systematic review	The aim of the present study was to carry out a systematic review of the scientific literature in order to obtain a deep insight into the factors that can influence the use of parks and the increase of PA in them, both from the point of view of design and perception of users of playgrounds
43	Identification of Effective Programs to Improve Access to and Use of Trails among Youth from Under-Resourced Communities: A Review.	Reed JA, Ballard RM, Hill M, Berrigan D.	2020	International Journal of Environmental Research and Public Health	review	The primary aim and objective of this review paper is to identify trail studies that may effectively promote and increase the use of trails among youth, especially those from under-resourced neighborhoods or communities. Three additional goals of the review include identifying: (1) Correlates of physical activity/trail use and features of transportation systems and/or built environment and land use destinations, that may inform and support the planning and implementation of programs to promote trail use among youth, (2) benefits associated with trail use, and (3) barriers to trail use.
44	Perceived Safety Influencing Active Travel to School-A Built Environment Perspective	Wangzom D, White M, Paay J.	2023	International Journal of Environmental Research and Public Health	narrative review	This may be because the research evidence lacks implementable results to improve active school travel. The active school travel research may require an evidence base for implementable intervention and this may be possible by adopting a future area of research to increase children walking/cycling to school. This paper identifies research

						gaps and proposes a way forward for a new research perspective that may be feasible to implement and may enhance active school travel.
45	Effectiveness of active school transport interventions: a systematic review and update.	Larouche R, Mammen G, Rowe DA, Faulkner G	2018	BMC Public Health	systematic review	We also aimed to review the literature on the moderators and mediators of AST interventions
46	Impact of public smoking bans on children's exposure to tobacco smoke at home: a systematic review and meta-analysis.	Nanninga S, Lhachimi SK, Bolte G.	2018	BMC Public Health	systematic review	this review aims to determine the overall impact of public smoking bans on children's SHS exposure at home.
47	Promoting active travel to school: a systematic review	Pang B, Kubacki K, Rundle-Thiele S.	2017	BMC Public Health	systematic review	First, we aim to conduct a systematic literature review and analysis of AST interventions published between 2010 and 2016. Second, we compare the results of our review with Chillon et al. [10] to assess whether significant differences in theory use, measurement and design are evident between time periods. Third, we assess the extent of theory use for AST interventions reporting theory.
48	Systematic review of how Play Streets impact opportunities for active play, physical activity, neighborhoods, and communities.	Umstattd Meyer MR, Bridges CN, Schmid TL, Hecht AA, Pollack Porter KM	2019	BMC Public Health	systematic review	the primary aim of this study was to document how Play Streets impact opportunities for safe active play and physical activity for children and adolescents. Secondary aims were to describe neighborhood and community impacts and examine evaluation

						methods used in each study in order to inform future evaluation research as Play Streets continue to grow in popularity.
49	The effectiveness of healthy community approaches on positive health outcomes in Canada and the United States.	Williams-Roberts H, Jeffery B, Johnson S, Muhajarine N.	2016	social sciences	narrative synthesis	The purpose of this review was to evaluate the evidence for the effectiveness of the ten most common healthy community approaches on positive health outcomes in Canada and the United States. This bridges a gap in the literature about what is effective and informs future priorities for research to strengthen the evidence base.
50	A review of the effect of traffic-related air pollution around schools on student health and its mitigation	An F, Liu J, Lu W, Jareemit D	2021	Journal of Transport and Health	review	The aim of this study was to identify effective measures that could be taken to alleviate traffic-related pollution and ensure student health.
51	Built environment associates of active school travel in New Zealand children and youth: A systematic meta-analysis using individual participant data.	Ikeda E, Stewart T, Garrett N, Egli V, Mandic S, Hosking J, et al	2018	Transport and Health	meta analysis	Therefore, the aims of this study were: (1) to systematically identify New Zealand research that had measured ATS, distance to school, and the neighbourhood built environment in children and youth, (2) to collate data from identified studies and combine them in a consistent manner, and (3) to identify associations between ATS and built environment features across the combined dataset. In doing so, this study provides new, robust evidence that has greater statistical power and is more generalisable than the contributing standalone studies.

52	Influence of Neighborhood Built Environments on the Outdoor Free Play of Young Children: a Systematic, Mixed-Studies Review and Thematic Synthesis.	Gemmell E, Ramsden R, Brussoni M, Brauer M	2023	J Urban Health	a Systematic, Mixed-Studies Review	We synthesize quantitative, qualitative, and mixed methods evidence to address the questions: 1. What features or characteristics of neighborhood built environments influence outdoor free play for children, ages 6 and under? 2. What features or characteristics of neighborhood built environments act as motivators, facilitators, or barriers to outdoor free play for children, ages 6 and under? 3. How do neighborhood built environment influences on young children's outdoor free play differ across child gender, cultures, and geographies?
55	Systematic review of interventions for promoting active school transport.	Villa-González E, Barranco-Ruiz Y, Evenson KR, Chillón P.	2018	Preventive Medecine	systematic review	the main aim of this study was to update the previous review published in 2011 (Chillon et al., 2011), following the same methodology and addressing the quality and effectiveness of new studies detected in the more recent scientific literature from 2010 to 2016.
56	Adoption of Safe Routes to School in Canadian and the United States Contexts: Best Practices and Recommendations	Macridis S, García Bengoechea E.	2015	Journal oh school health	realist review	This article aims to identify safe route to school programs in canada and US at the state/province/city level to explore and understrand strategie to initiate the process ans to: uncover political contexts; identify SRTS goals ans approches; examine SRTS partenerships establishment; identify evaluation tools; and discuss benefits, challenges, and lessons learned;
57	Walking school buses as a form of active	Smith L, Norgate SH, Cherrett T, Davies N,	2015	Journal of School Health	review	Alongside this, to increase knowledge about the rigor of research methods

	transportation for children-a review of the evidence.	Winstanley C, Harding M.				used to investigate the role of WSBs in children's fitness related health, with a view to making recommendations for future studies.
58	The decline in active school transportation (AST): A systematic review of the factors related to AST and changes in school transport over time in North America.	Rothman L, Macpherson AK, Ross T, Buliung RN.	2018	Preventive Medecine	systematic review	A systematic review of the literature was conducted to identify correlates representing different social-ecological domains related to AST in North America and to discuss how changes in these correlates could impact school travel over time.
69	Neighbourhood speed limit and childhood obesity	Luo M, Li H, Pan X, Fei T, Dai S, Qiu G, et al	2021	Obesity Reviews	sytematic review	This study aimed to conduct a systematic review of the association between speed limit and weight-related behaviours or weight status among children and adolescents.
71	Social and physical environmental correlates of independent mobility in children: a systematic review taking sex/gender differences into account	Marzi I, Demetriou Y, Reimers AK.	2018	International Journal of Healh Geographics	systematic review	This systematic review aims to provide an overview of socioecological correlates of CIM with a particular focus on differences between boys and girls and categorized by different CIM types.
81	Independent Mobility and Mode Choice for School Transportation: A Review and Framework for Future Research	Mitra R.	2013	Transport Reviews	scoping review	The paper has three objectives: (1) to evaluate the major theoretical approaches used by researchers to study and explain walking/cycling behaviour in general, and AST in particular; (2) to review evidence of the potential influences on school travel outcomes and (3) to outline a conceptual

						framework of the relationship between the environment, household travel constraints and school travel outcomes, in order to guide future research on school transportation behaviour, particularly in North America.
82	A systematic review of the risk factors and interventions for the prevention of playground injuries	Richmond SA, Clemens T, Pike I, Macpherson A	2018	Canadian Journal of Public health	systematic review	The primary objectives of this work, therefore, were to (1) identify the risk and/or protective factors associated with playground injuries among children less than 18 years of age and (2) identify interventions/programs/policies aimed at preventing playground-related injuries among children less than 18 years of age. Secondary objectives include a summary of the data to reflect alignment with current playground standards.
86	The impacts of unstructured nature play on health in early childhood development: A systematic review	Dankiw K, Tsiros M, Baldock K, Kumar S.	2020	plos one	systematic review	The purpose of this systematic review was to identify the impact of participating in nature play on health and developmental outcomes in children aged 2–12 years.
88	Physical activity and social connectedness interventions in outdoor spaces among children and youth: a rapid review.	Wray A, Martin G, Ostermeier E, Medeiros A, Little M, Reilly K, et al.	2020	Health Promotion and Chronic Disease Prevention in Canada	rapid review	Our aim was to identify interventions that have the greatest positive effects on childhood physical activity and social connectedness. This review critically synthesizes interventions in outdoor spaces that have outcome measures for physical activity or social connectedness among children and youth (aged 19 years and less) from Australia, Canada,

						Europe (including Turkey), New Zealand and the United States of America (USA).
89	All the fun stuff, the teachers say, "that's dangerous!"' Hearing from children on safety and risk in active play in schools: a systematic review	Jerebine A, Fitton-Davies K, Lander N, Eyre ELJ, Duncan MJ, Barnett LM	2022	International Journal of Behavioral Nutrition and Physical Activity	systematic review	This systematic review of qualitative literature aimed to synthesise research conducted with children that explored how safety and risk shape active play during recess in elementary and middle school. Specifically, this review sought to identify how risk and safety afford or constrain children's play in schools, and how these factors serve to motivate or discourage children from playing actively. A secondary aim was to develop a model from the findings to guide efforts in schools to optimise children's play opportunities during recess.
90	'Children are precious cargo; we don't let them take any risks!': Hearing from adults on safety and risk in children's active play in schools: a systematic review	Jerebine A, Fitton-Davies K, Lander N, Eyre ELJ, Duncan MJ, Barnett LM	2022	International Journal of Behavioral Nutrition and Physical Activity	qualitative systematic review	this qualitative systematic review aimed to examine adult (e.g., parent, teacher, yard supervisor, principal, administrator) perspectives on safety and risk in children's active play during recess in elementary and/or middle schools.
91	The value of (pre)school playgrounds for children's physical activity level: a systematic review.	Broekhuizen K, Scholten AM, de Vries SI.	2014	International Journal of Behavioral Nutrition and Physical Activity	review	the aim of this review paper is to present an overview of the existing evidence on the value of (pre)school playgrounds for children's health in terms of physical activity, cognitive and social outcomes. Further, we aimed to identify which playground characteristics are most effective, and for which subgroups of children effects are most distinct.

93	Increasing Nature Connection in Children: A Mini Review of Interventions.	Barrable A, Booth D.	2020	frontiers in Psychology	mini review	the review aims to identify and summarize the key points of interventions that promote a connection to nature in people <18 years of age, and provide some guidelines for future research.
94	Effects of Nature (Greenspace) on Cognitive Functioning in School Children and Adolescents: a Systematic Review.	Vella-Brodrick DA, Gilowska K.	2022	Educational Psychology Review	systematic review	this review will: (1) systematically evaluate the recent body of experimental and quasi-experimental studies examining the effects of nature (greenspace) on cognitive functioning in children and adolescents; (2) investigate the underlying processes involved in possible effects of nature on cognitive performance as well as consider the adequacy of different theoretical models to explain any effects; and (3) examine whether the effects of nature exposure differ according to age. We will also explore the duration and type of nature intervention (active or passive) to determine whether these intervention characteristics influence outcomes.
95	Effects of Enactment of Legislative (Public) Smoking Bans on Voluntary Home Smoking Restrictions: A Review	Monson E, Arsenault N	2017	Nicotine & Tobacco Research	systemtic review	This review aims to determine the overall effect of the enactment of legislative (public) smoking bans or restrictions on voluntary smoking restrictions in private residences as well as to evaluate the current status of the literature and the strength of evidence for and against diffusion and social displacement models.

96	Explaining Mechanisms That Influence Smoke-Free Implementation at the Local Level: A Realist Review of Smoking Bans	Mlinarić M, Hoffmann L, Kunst AE, Schreuders M, Willemsen MC, Moor I, et al.	2019	Nicotine & Tobacco Research	realist review	we aim to translate existing evidence into practical recommendations on how to improve SF (outdoor) implementation within European municipalities.
98	Promoting activity participation and well-being among children and adolescents: a systematic review of neighborhood built-environment determinants.	Nordbø ECA, Nordh H, Raanaas RK, Aamodt G	2020	JBI Evidence Synthesis	systematic review	The objective of this review was to identify, evaluate, and synthesize the findings on built-environment determinants and their relation to participation in different domains of activities, including PA, recreational and social activities, and wellbeing among children and adolescents from a broader public health perspective. In particular, the objective was to identify which built-environment determinants seem to promote participation in activities and wellbeing in childhood and adolescence.
101	Child-Friendly Environments-What, How and by Whom?	Jansson M, Herbert E, Zalar A, Johansson M	2022	sustainability	review	The aim of this study is to analyze and synthesize the concept of child-friendly environments and its use within the research literature, in order to reach a more in-depth understanding of the socio-physical qualities and actors supporting its realization. The study is based on the following research questions: 1. In what contexts have CFE been studied up to the year 2020? 2. What actors and activities are involved in CFE? 3. What factors are strong indicators for CFE? 4. What socio-physical factors have been found to be important for realizing CFE?

102	Perceived barriers to children's active commuting to school: a systematic review of empirical, methodological and theoretical evidence	Lu W, McKyer EL, Lee C, Goodson P, Ory MG, Wang S.	2014	International Journal of Behavioral Nutrition and Physical Activity	systematic review	we aimed to 1) examine research on perceived barriers to ACS, 2) identify different types and measures of perceived barriers reported by researchers, 3) assess the methodological quality of empirical studies on perceived barriers to ACS, and 4) evaluate the level of theory utilization in the studies, i.e., to what extent theory was used and how the construct of perceived barriers was conceptualized and operationalized.
103	Examining the Implementation of Play Streets: A Systematic Review of the Grey Literature	Bridges CN, Prochnow TM, Wilkins EC, Porter KMP, Meyer MRU	2020	Journal of Public Health Management and Practice	systematic review of the grey literature	The aim of this systematic review of the grey literature is to examine and describe what is known about implementing Play Streets using the RE-AIM (Reach, Effectiveness, Adoption, Implementation, and Maintenance) framework, which is widely used in implementation research
104	Economics of Interventions to Increase Active Travel to School: A Community Guide Systematic Review	Jacob V, Chattopadhyay SK, Reynolds JA, Hopkins DP, Morgan JA, Brown DR, et al.	2021	American Journal of Preventive Medicine	review	This study asks what it costs to implement ATS interventions and what the economic benefits are that result from the intervention. Do the economic benefits due to intervention exceed the cost to implement? The objective of this study is to synthesize the economic evidence for the cost and benefit of these programs.
